# Optimal DMD Koopman Data-Driven Control of a Worm Robot

**DOI:** 10.3390/biomimetics9110666

**Published:** 2024-11-01

**Authors:** Mehran Rahmani, Sangram Redkar

**Affiliations:** The Polytechnic School, Ira Fulton School of Engineering, Arizona State University, Mesa, AZ 85212, USA; sangram.redkar@asu.edu

**Keywords:** data-driven, DMD method, Koopman theory, LQR, worm robot

## Abstract

Bio-inspired robots are devices that mimic an animal’s motions and structures in nature. Worm robots are robots that are inspired by the movements of the worm in nature. This robot has different applications such as medicine and rescue plans. However, control of the worm robot is a challenging task due to the high-nonlinearity dynamic model and external noises that are applied to that robot. This research uses an optimal data-driven controller to control the worm robot. First, data are obtained from the nonlinear model of the worm robot. Then, the Koopman theory is used to generate a linear dynamic model of the Worm robot. The dynamic mode decomposition (DMD) method is used to generate the Koopman operator. Finally, a linear quadratic regulator (LQR) control method is applied for the control of the worm robot. The simulation results verify the performance of the proposed control method.

## 1. Introduction

Bio-inspired robots are a class of robots designed by emulating the movement and structural characteristics of animals found in nature. Researchers study various animals, analyzing their unique forms of locomotion and adapting these biological mechanisms to robotic designs. One prominent example of this is the worm robot, which replicates the movement patterns of worms in nature [1,2,3,4,5]. This bio-inspired robot has garnered significant interest due to its versatility and potential applications, particularly in fields such as medicine, exploration, and disaster rescue operations. Worm robots can navigate through complex and harsh environments that would be difficult for traditional robots to traverse. They are especially useful in medical applications, where they can maneuver through the human body for minimally invasive surgeries or deliver targeted treatments. In rescue operations, their ability to crawl through narrow, uneven spaces allows them to access areas that might be otherwise unreachable, making them invaluable in situations like natural disasters or collapsed structures. However, controlling such robots presents unique challenges. Their soft, flexible bodies require sophisticated control methods to ensure precise movement and navigation through unpredictable environments. The dynamic nature of their movement, combined with external factors like varying terrain and obstacles, makes developing effective control algorithms for worm robots a complex task. Researchers are continually working to enhance these control strategies, focusing on stability, adaptability, and efficiency to improve the performance of worm robots in real-world applications.

Several control methods have been employed to control worm robots along desired trajectories. One such method is the robust adaptive controller proposed in [6], which integrates multiple control strategies to enhance performance. The approach begins with the application of sliding mode control to ensure robustness against external disturbances. Following this, a fractional proportional-integral-derivative (PID) controller is incorporated to improve the tracking performance. The bat algorithm is utilized to fine-tune the parameters of this hybrid controller, optimizing the control system for the specific dynamics of the worm robot. Another notable control method highlights the crucial role of friction in the generation and control of locomotion in worm robots, leveraging the concept of a controlled subspace [7]. This method introduces a simulation-based technique for creating and executing feedback control schemes, enabling the robot to achieve both forward and backward locomotion. Based on this analysis, the control strategy is demonstrated to be effective for the robot’s locomotion in various directions. In addition to these approaches, other controllers have been employed for worm robot control, such as hybrid neural network-based integral sliding mode control [8] and neural central pattern generator (CPG) approaches [9]. While these controllers rely heavily on model-based control methods or artificial intelligence techniques to govern worm robot behavior, they do not utilize data-driven control methods. This gap can be addressed by the Koopman theory, a powerful framework for controlling complex nonlinear dynamic systems. The Koopman operator offers a transformative data-driven approach for analyzing and controlling nonlinear systems characterized by high-dimensional dynamics. This theory facilitates the control of nonlinear systems by leveraging data-driven methods to linearize them in higher-dimensional spaces. In [10], the Koopman theory is proposed as a robust tool for controlling worm robots, offering significant advantages over traditional model-based techniques. The data-driven nature of Koopman theory allows it to capture the intrinsic complexities of nonlinear systems, enabling more precise and efficient control.

Moreover, in [11], a Koopman-based system identification technique is detailed, highlighting its application in designing model predictive controllers (MPCs) for soft robots. The study demonstrates the development of three Koopman-based MPCs for a pneumatic soft robot arm, with their performance evaluated across multiple real-world trajectory-tracking tasks. The results show that these Koopman-based controllers achieve an average tracking error more than three times lower than that of a benchmark MPC based on a linear state-space model of the same system. This showcases the significant potential of the Koopman approach in enhancing control accuracy for soft robots, making it an ideal candidate for further exploration in worm robot control. Sun et al. [12] propose an MPC method for the closed-loop motion control of a tendon-driven continuum robot with two bending degrees of freedom, leveraging the constant curvature model for kinematic representation. Their experiments demonstrate that the MPC-based controller significantly reduces position error compared to an open-loop controller, highlighting its effectiveness in achieving precise trajectory tracking. Wang et al. [13] address the challenges of accurately controlling continuum robots by modeling their complex nonlinear dynamics and employing a model predictive control (MPC) strategy to achieve real-time control of the target angle. Their approach involves linearizing and discretizing the nonlinear system, demonstrating that MPC significantly outperforms traditional PD control methods by effectively driving the target angle to its desired value quickly and with satisfactory accuracy.

The LQR (linear quadratic regulator) control method is a robust optimal control approach that enhances trajectory tracking performance and ensures greater system stability. In this study, the system dynamics are assumed to be unknown in addressing the finite-horizon linear quadratic regulation problem [14]. A limited number of input-state variables can still provide critical information about the system’s behavior, provided that the input is sufficiently stimulating and of a high-enough order. To determine the optimal control law, the problem is formulated and solved as a semidefinite program using available data. A neural network is employed in [15] to construct the Koopman operator, which is then applied to develop a linear dynamic model for robot manipulators with 7 degrees of freedom (DoFs). An LQR controller is subsequently designed to minimize tracking errors and improve control accuracy. Fan and Xiong [16] address the linear quadratic optimal control problem for unknown stochastic-parameter linear systems through reinforcement learning methods. They propose a model-based value iteration algorithm that leverages the second moments of random system matrices, proving its convergence using the contraction mapping theorem. Additionally, a normalized model-free value iteration algorithm is introduced for scenarios where information about random system matrices is unavailable, demonstrating that normalization reduces convergence errors and allows for the learning of an approximate optimal control policy applicable to any distribution of the random system matrices without requiring an initial stabilizing control policy. Elkhatem et al. [17] explore the stabilization of unmanned aerial vehicles (UAVs) against wind and environmental disturbances using LQR controllers. They propose an innovative method for automatically adjusting the weighting matrices based on state variables and a designer-determined preference factor to enhance robustness against disturbances. Experimental results demonstrate that both LQR and LQR with a PI controller effectively stabilize the quadrotor, with the LQR-PI method achieving superior tracking performance and robustness.

Dynamic mode decomposition (DMD) and Koopman control are powerful techniques used in the analysis and control of dynamical systems, particularly in the context of nonlinear systems [18,19,20,21]. DMD is a data-driven method that decomposes complex systems into a set of modes, which are temporally coherent structures in the data. By applying DMD to time series data, one can extract the dominant modes of a system’s dynamics, allowing for a clearer understanding of its behavior. The key idea behind DMD is to represent the dynamics of a system in terms of its eigenvalues and eigenvectors. Each mode corresponds to a spatial pattern of behavior in the system, while the associated eigenvalues provide insights into the growth or decay rates of these patterns over time. This decomposition is particularly useful for analyzing systems with complex, nonlinear dynamics, as it simplifies the representation of the underlying behavior. Koopman control theory builds upon the DMD framework by leveraging the concept of the Koopman operator, which is an infinite-dimensional linear operator that describes the evolution of observable functions of a dynamical system [22,23]. The key advantage of the Koopman operator is that it enables linear control techniques to be applied to nonlinear systems by transforming the problem into a higher-dimensional space where the dynamics can be treated linearly. By identifying a suitable set of observables, one can approximate the Koopman operator, thus allowing the design of controllers based on linear control strategies such as LQR. This approach not only simplifies the control design but also provides stability guarantees and improved performance in controlling complex, nonlinear systems [24,25]. The combination of DMD and Koopman control provides a robust framework for analyzing and controlling nonlinear dynamical systems by leveraging data-driven techniques to extract modes and utilizing the linearization of dynamics in an extended observable space for effective control design.

The most crucial aspect of utilizing the Koopman operator is how it is derived. Various methods can be employed to compute the Koopman operator, including techniques such as neural networks [15,26] and the dynamic mode decomposition (DMD) approach [27]. Each method provides distinct advantages in transforming nonlinear system dynamics into a linear representation, facilitating the design of linear controllers. The primary contributions of this research are outlined as follows:

1. A novel optimal data-driven controller is proposed to control the movement and behavior of a worm robot.
2. The study introduces and models the complex nonlinear dynamics of a worm robot.
3. The Koopman theory is applied to transform the nonlinear dynamics into a linear framework, enabling more straightforward controller design.
4. The DMD method is employed to generate the Koopman operator, providing a data-driven approach to linearize the robot’s dynamics.
5. An LQR controller is utilized to regulate the linearized dynamic model of the worm robot, enhancing the precision of trajectory tracking and overall control performance.

The structure of the paper is organized as follows: Section 2 introduces the nonlinear dynamic model of the worm robot, laying the groundwork for the control strategy. Section 3 discusses the fundamentals of Koopman theory and its relevance to this study. Section 4 details the DMD method used to generate the Koopman operator. Section 5 describes the design and implementation of the LQR control method. Section 6 presents the simulation results, highlighting the effectiveness of the proposed control strategy. Finally, Section 7 concludes the paper by summarizing the findings and discussing potential future directions for research.

## 2. Worm Robot Structure

**Figure 1:**
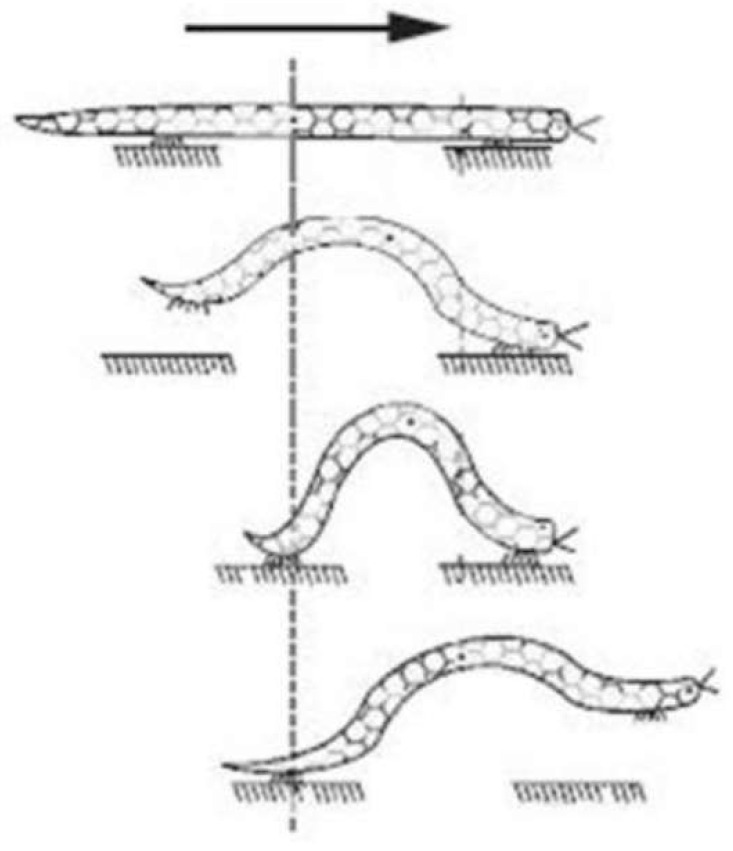
Worm robot motion in nature [28].

**Figure 2:**
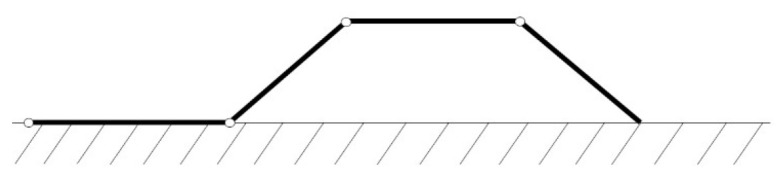
Worm robot mechanism.

The worm robot is inspired by the motion of the worm in nature. The type of worm motion considered in this research is inchworm locomotion, which involves a distinctive movement pattern characterized by a series of extensions and contractions. This method of locomotion allows the robot to replicate the fluid, wave-like motion seen in natural inchworms, facilitating efficient movement across various surfaces. Figure 1 shows a worm motion in nature. Therefore, if the motion changes to a mechanism, the robot will be created. The motion of the worm can be demonstrated as a mechanism in Figure 2. The scholars obtained the following nonlinear dynamics equations of the mechanism in Figure 2 [27]:

(1) $$M\left(\theta \right)\ddot{\theta }+V\left(\theta \right){\dot{\theta }}^{2}+G\left(\theta \right)=K\tau +\lambda F_{R}$$

where $M(\theta )∊R^{4\times 4}$, $V(\theta )∊R^{4\times 4}$, $G(\theta )∊R^{4\times 1}$, and $K∊R^{4\times 1}$ are the mass matrix, centrifugal coefficient matrix, gravity vector, and subtraction matrix. Also, $F_{R}∊R^{4\times 1}$ represents friction forces on the tip of the last link that apply from the ground. The detailed process of obtaining Equation (1) can be observed in [29].

$$M\left(\theta \right)=\frac{ml^{2}}{6}\left[\begin{array}{ccc} 20 & 15c_{12} & \begin{array}{cc} 9c_{13} & 3c_{14} \end{array}\\ 15c_{12} & 14 & \begin{array}{cc} 9c_{23} & 3c_{24} \end{array}\\ \begin{array}{c} 9c_{13}\\ 3c_{14} \end{array} & \begin{array}{c} 9c_{23}\\ 3c_{24} \end{array} & \begin{array}{c} \begin{array}{cc} 8 & 3c_{34} \end{array}\\ \begin{array}{cc} 3c_{34} & 2 \end{array} \end{array} \end{array}\right]$$

$$V\left(\theta \right)=\frac{ml^{2}}{6}\left[\begin{array}{ccc} 0 & 15s_{12} & \begin{array}{cc} 9s_{13} & 3s_{14} \end{array}\\ -15s_{12} & 0 & \begin{array}{cc} 9s_{23} & 3s_{24} \end{array}\\ \begin{array}{c} -9s_{13}\\ -3s_{14} \end{array} & \begin{array}{c} -9s_{23}\\ -3s_{24} \end{array} & \begin{array}{c} \begin{array}{cc} 0 & 3s_{34} \end{array}\\ \begin{array}{cc} -3s_{34} & 0 \end{array} \end{array} \end{array}\right]$$

$$G\left(\theta \right)=\frac{mgl}{2}\left[\begin{array}{c} 7cos\theta_{1}\\ 5cos\theta_{2}\\ \begin{array}{c} 3cos\theta_{3}\\ cos\theta_{4} \end{array} \end{array}\right]$$

$$\lambda =\left[\begin{array}{ccc} 1 & -1 & \begin{array}{cc} 0 & 0 \end{array}\\ 0 & 1 & \begin{array}{cc} -1 & 0 \end{array}\\ \begin{array}{c} 0\\ 0 \end{array} & \begin{array}{c} 0\\ 0 \end{array} & \begin{array}{c} \begin{array}{cc} 1 & -1 \end{array}\\ \begin{array}{cc} 0 & 1 \end{array} \end{array} \end{array}\right]$$

where $\theta_{i}$ is the angle of the *ith* joint. Also, $c_{ij}=\cos\left(\theta_{i}-\theta_{j}\right)$ and $s_{ij}=sin(\theta_{i}-\theta_{j})$. *m* is the mass of each link and *l* is the length of each link.

## 3. Koopman Theory

The Koopman operator theory offers a powerful framework for analyzing and solving nonlinear dynamical systems by transforming them into a higher-dimensional linear system. Specifically, it proposes that to capture the dynamics of a nonlinear system, the system’s initial form must be mapped into an infinite-dimensional state space. In this higher-dimensional space, the original nonlinear dynamics can be represented and studied as a linear system, which significantly simplifies analysis and control.

This transformation is possible because the Koopman operator acts on the space of observable functions, rather than the state variables themselves. These observables can include nonlinear functions of the system’s states, allowing the dynamics to be expressed linearly in terms of these observables, even though the system remains nonlinear in its original form. The main advantage of this approach is that linear systems are typically much easier to work with, especially in the context of control and prediction. By applying this approach, one can analyze and control complex nonlinear systems using the rich set of tools developed for linear systems, leading to more efficient and effective solutions in various engineering and scientific fields [30,31,32,33].

(2) $$z_{k+1}=F\left(z_{k}\right)$$

In dynamical systems, the function *F* is defined as follows:

(3) $$F\left(z\left(t_{0}\right)\right)=z\left(t_{0}\right)+\int_{t_{0}}^{t_{0}+t}f\left(z\left(\tau \right)\right)d\tau$$

The Koopman operator theoretical approach provides a powerful framework for transforming a finite-dimensional nonlinear system into a linear system, but in an infinite-dimensional space. Specifically, the Koopman operator lifts the dynamics of the system into an infinite-dimensional function space, where it acts on observables—real-valued functions that measure certain aspects of the system’s state. Let *g* be an observable, which is a scalar measurement function; the Koopman operator *K* acts on it by advancing it in time as follows:

(4) Kg=g∘F

In other words, the Koopman operator propagates the observable function *g* forward according to the flow of the system defined by *F*. For continuous-time systems, the dynamics of the system can be smoothly described through the observable function *g*. The evolution of *g* over time is governed by the following:

(5) $$\frac{d}{dt}g\left(z\right)=Kg\left(z\right)=\nabla g\left(z\right).f\left(z\right)$$

Here, $\nabla g\left(z\right)$ represents the gradient of the observable with respect to the state *z*, and *f*(*z*) is the system’s vector field. This equation highlights how the Koopman operator facilitates the linear evolution of the observable function *g* within the infinite-dimensional space, making it useful for analysis. However, while the infinite dimensions of the Koopman operator are crucial for capturing the complete system dynamics, they also pose challenges for practical computation and representation. To address this, applied Koopman analysis typically focuses on approximating the operator in a lower-dimensional subspace. This is achieved by considering a finite set of observables, which span an invariant subspace of the Koopman operator.

When the Koopman operator is restricted to such a subspace, it can be represented as a finite-dimensional matrix. This allows for more tractable computation while still retaining key features of the original system. The Koopman eigenfunctions, denoted by $\varphi \left(z\right)$, play a central role in this approximation. Each eigenfunction is associated with a corresponding eigenvalue λ, satisfying the following relationship:

(6) $$\lambda \varphi \left(z\right)=\varphi \left(F\left(z\right)\right)$$

In continuous time, the evolution of the Koopman eigenfunction $\varphi \left(z\right)$ is governed by the following:

(7) $$\frac{d}{dt}\varphi \left(z\right)=\lambda \varphi \left(z\right)$$

This equation shows that the Koopman eigenfunction evolves exponentially over time, with the rate of growth determined by its associated eigenvalue λ.

**Figure 3:**
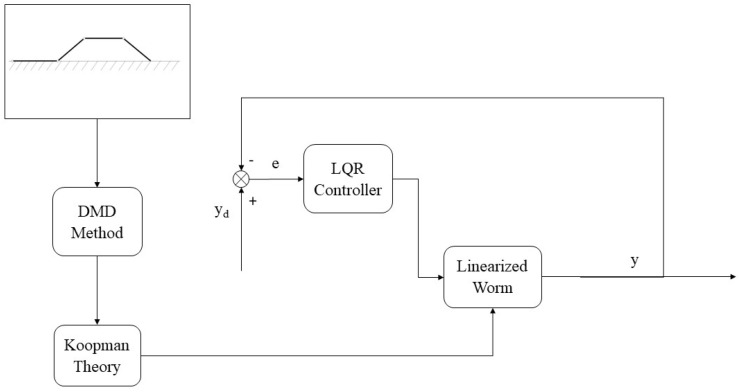
The proposed control method diagram.

To approximate the Koopman operator in practice, we rely on finite-dimensional approximations. One widely used technique is dynamic mode decomposition (DMD), which estimates the Koopman operator by constructing a low-rank approximation based on snapshots of the system’s state over time. DMD provides a practical way to approximate the infinite-dimensional Koopman operator by capturing dominant modes of the system, allowing for efficient analysis and control design. The proposed control method is illustrated in the block diagram (Figure 3), which integrates the Koopman operator framework with the control strategy to handle nonlinear systems effectively.

## 4. DMD Method

The DMD method is a powerful data-driven technique used to approximate the Koopman operator, a linear operator that governs the evolution of nonlinear dynamical systems in an infinite-dimensional space. DMD extracts dynamic information from time series data by decomposing the observed data into modes associated with distinct temporal frequencies and growth rates. It can be understood as a numerical procedure to find the best-fit linear system to a set of data, despite the underlying dynamics being nonlinear. The method is especially valuable in fluid dynamics, control systems, and other fields that deal with complex, nonlinear phenomena.

In the context of obtaining the Koopman operator, DMD helps identify a finite-dimensional approximation of this operator from the data. Essentially, it operates by approximating the evolution of observables (functions of the system state) rather than the state itself. The data collected from the system are arranged into matrices, and a best-fit linear model is derived that governs the progression of these observables. By doing so, DMD provides a practical means to approximate the infinite-dimensional Koopman operator, thus enabling the use of linear analysis tools for otherwise nonlinear systems. This makes DMD a critical technique in modern control theory, especially for model reduction, prediction, and the control of complex systems.

The dynamic mode decomposition (DMD) method is a powerful technique used to approximate the Koopman operator, which allows for the decomposition of complex, nonlinear systems into a linear framework. This approximation can be written as follows:

(8) $$Z^{\prime }\approx AZ$$

where $Z^{\prime }$ is the time-shifted matrix of Z, representing the system’s state at a future time step, and *A* is the matrix that approximates the dynamics of the system.

Matrix Z is defined as a collection of snapshots:

$$Z=\left[\begin{array}{ccc} z_{1} & z_{2} & \ldots \ldots . \end{array}\right]$$

These snapshots are system states observed at discrete time intervals. To determine matrix *A*, we use the following equation:

(9) $$A=Z^{\prime }Z^{+}$$

where $Z^{+}$ represents the Moore–Penrose pseudoinverse of Z. This pseudoinverse is used because Z is often not square, and the pseudoinverse provides a way to solve the least-squares problem for over- or under-determined systems. Calculating *A* directly, especially for large systems, can be computationally expensive due to the high dimensionality involved. To reduce the computational complexity, singular value decomposition (SVD) is applied to the matrix of snapshots, which allows us to approximate Z with reduced rank. SVD is given by the following [32]:

(10) $$Z\approx U\Sigma V^{*}$$

Here, $U∊R^{n\times r}$, $\Sigma ∊R^{r\times r}$, $V∊R^{n\times r}$. The symbol * denotes the conjugate transpose, and *r* represents the reduced rank that approximates *Z*. By truncating the rank, we significantly reduce the size of the system, capturing the most important features of the dynamics while maintaining computational efficiency.

The eigenvectors Φ, which provide insight into the system’s modes, can be defined as follows:

(11) $$\Phi =Z^{\prime }V\Sigma^{-1}W$$

where W denotes the eigenvectors of the dynamic system with complete rank. These eigenvectors represent the dominant modes of the system and are crucial for understanding its dynamic behavior.

If we assume that λ represents the eigenvalue associated with a particular mode, then the Koopman operator K satisfies the following relationship:

(12) KW=λW

This equation captures the linear evolution of the system’s modes under the action of the Koopman operator. Finally, the linearized dynamic model of the system can be expressed as follows:

(13) $$\frac{d}{dt}y=Ky+Bu$$

where y is the state of the system, *K* is the Koopman operator that governs the linear evolution of the system’s modes, and *B* is the input matrix that represents how external inputs *u* affect the system. This formulation provides a clear link between the nonlinear system and its linear representation using the Koopman operator, enabling efficient analysis and control design. By leveraging DMD and Koopman operator theory, we can transform complex, nonlinear dynamics into a linear form that is easier to analyze, compute, and control, particularly for high-dimensional systems.

## 5. LQR Control Method

The linear quadratic regulator (LQR) is a fundamental control strategy used in optimal control theory. It seeks to find the best possible control input that minimizes a quadratic cost function, which typically balances two competing objectives: minimizing the state deviation from a desired reference and minimizing the control effort. The LQR controller achieves this by solving the Riccati equation to compute a feedback gain matrix that drives the system to stability. This feedback gain matrix is multiplied by the state vector to produce the control input, ensuring that the system behaves in a desired manner over time.

One of the key benefits of LQR is its ability to provide optimal performance while accounting for system dynamics. Unlike simple proportional control methods, LQR considers the entire system model and adjusts the control signal to minimize both transient and steady-state errors. It also balances performance and energy efficiency by penalizing large control inputs, making it particularly useful in systems where control effort needs to be minimized, such as robotics and aerospace applications. Additionally, LQR can be easily extended to multivariable systems, making it versatile for a wide range of applications. LQR can be effectively applied to nonlinear systems after linearizing them using Koopman theory. The Koopman operator allows for the representation of a nonlinear system in a higher-dimensional linear space by mapping the system’s state into an observable space. Once the nonlinear system is expressed in this linear form, the standard LQR technique can be employed to design an optimal controller. By utilizing Koopman-based linearization, the benefits of LQR—such as minimizing a quadratic cost function and ensuring optimal performance—can be extended to complex, nonlinear systems. This approach enables the design of efficient control strategies for systems where traditional linearization methods may fall short, offering more accurate and robust control performance.

Making a state feedback controller that minimizes the target function is the aim of the LQR design challenge. The cost function is defined as follows:

(14) $$J=\int_{0}^{\infty }\left(y^{T}Qy+u^{T}Ru\right)dt$$

where *Q* and *R* are the weight matrices. The feedback control law that reduces the value of the cost function is as follows:

(15) u=−Cy

where *C* is denoted as follows:

(16) $$C=R^{-1}B^{T}P$$

*P* is found by solving the continuous-time Riccati algebraic problem.

(17) $$K^{T}P+PK+Q-PBR^{-1}B^{T}P=0$$

## 6. Simulation Results

The simulation results provide a comprehensive assessment of the control performance achieved through the Koopman–LQR approach. By leveraging the Koopman operator to linearize the worm robot’s complex nonlinear dynamics, the control strategy effectively transforms the system into a manageable linear model, allowing for the application of the LQR controller. The simulation began with the angular velocities of all joints initialized to zero, and the initial joint positions set to $\theta_{0}=\left\{\frac{\pi }{4},\frac{\pi }{2},0,0\right\}$. The mass and length of each link were both set to one unit, providing a simplified yet meaningful representation of the robot’s dynamics. *y_d_* is the desired tracking, which is equal to zero.

**Figure 4:**
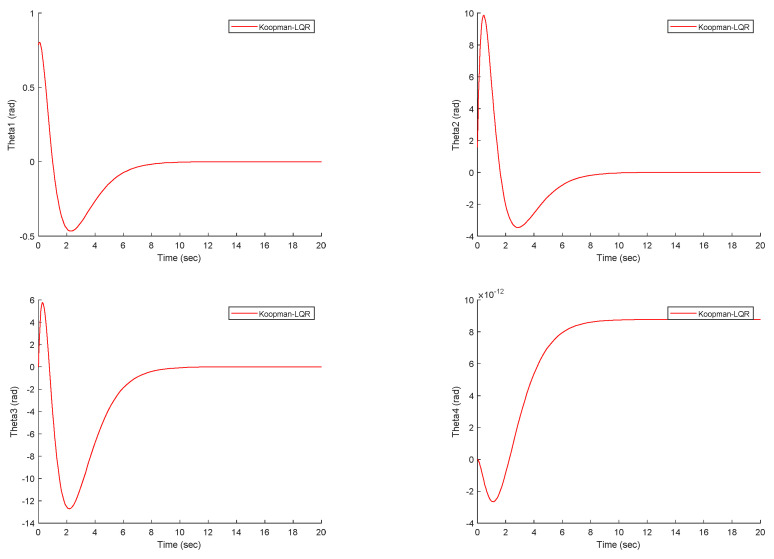
The position tracking of the worm robot joints under the proposed controller.

**Figure 5:**
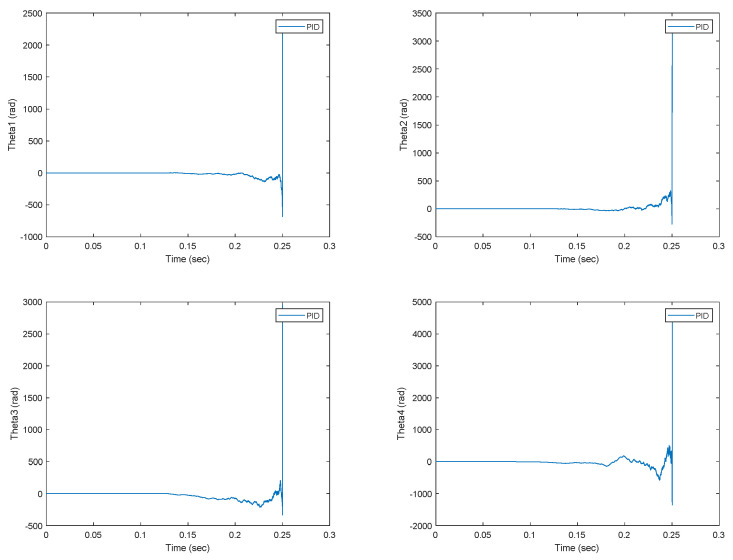
The position tracking of the worm robot joints under the PID controller.

The DMD input data are generated from the nonlinear dynamics of the worm robot using the ode45 solver in MATLAB. In Figure 4, the position tracking of the robot joints demonstrates that the proposed Koopman–LQR controller achieves high precision across all joints. The joint angles smoothly follow their reference trajectories, with minimal deviation. The notable 46% overshoot observed in Theta1, although substantial, remains within acceptable limits given the nonlinear nature of the original system. This indicates a good balance between system responsiveness and stability. The controller quickly mitigates the overshoot, ensuring that the joints settle into their desired positions with minimal oscillation. This is particularly important for systems with flexible links like the worm robot, where excessive oscillations could lead to mechanical stress or failure. Additionally, the position tracking of the remaining joints shows even better performance, with negligible overshoot or steady-state error, further reinforcing the effectiveness of the Koopman-LQR control strategy in handling dynamic complexity. These results highlight the robustness of the control method in compensating for the nonlinearities inherent in the system. The results in Figure 5, which present the tracking performance of the worm robot under the proportional-integral-derivative (PID) controller, demonstrate that the PID controller is not suitable for controlling the worm robot’s dynamics. The key issue is that the PID controller is designed for systems with linear or near-linear dynamics, whereas the worm robot exhibits highly nonlinear behavior. As a result, the PID controller struggles to maintain stability and precision in tracking the desired trajectories, especially in the presence of nonlinearity and complex interactions between the robot’s joints. By attempting to control the system before applying the Koopman operator to derive a linear data-driven dynamic model, the limitations of traditional linear control methods, such as PID, become evident. These limitations include oscillations in the tracking error and the inability to handle the dynamic coupling between the joints. This makes the PID controller ineffective, as it does not account for the robot’s nonlinear dynamics. Thus, using the Koopman operator to first approximate a linear model of the nonlinear system provides a more suitable framework for control design. This data-driven approach captures the underlying system dynamics more accurately, allowing for the application of advanced linear control strategy such as LQR, which is more robust to the complexities of the worm robot. Therefore, Figure 5 emphasizes the necessity of applying a Koopman-based approach for better control, stability and performance.

**Figure 6:**
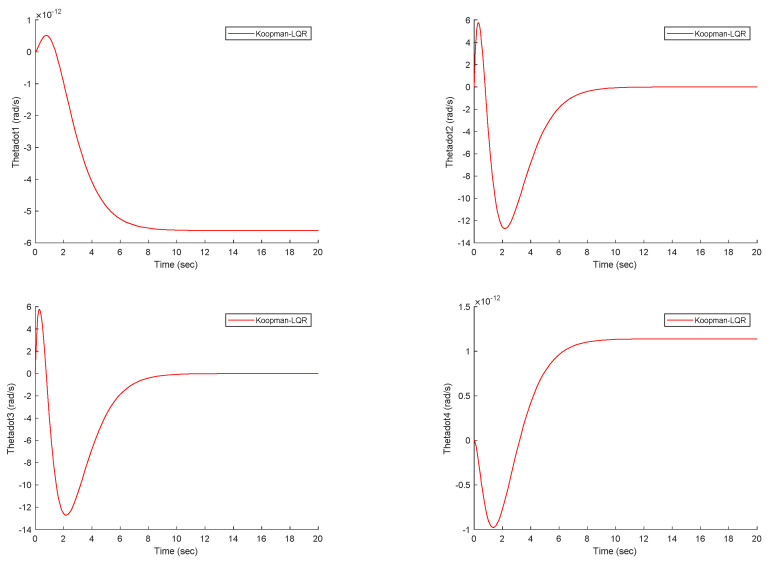
The velocity of the worm robot joints under the proposed controller.

Figure 6 illustrates the velocities of the robot joints under the Koopman–LQR controller. The velocity responses are smooth, with no noticeable high-frequency oscillations or instability, which would indicate chattering or poor control performance. The steady convergence of the joint velocities to their desired values demonstrates that the controller efficiently manages both transient and steady-state behaviors. Furthermore, the absence of sudden spikes or disturbances in the velocity plots shows that the proposed control method not only regulates the robot’s positions effectively but also ensures smooth motion transitions between different operating states. The combination of position and velocity responses highlights the advantage of using Koopman theory to linearize the system. By capturing the underlying dynamic modes of the robot and approximating the nonlinear system with an equivalent linear representation, the control design simplifies the otherwise complex task of controlling a highly nonlinear system. This linearized model allows the LQR controller to function optimally, leading to efficient and precise control.

**Figure 7:**
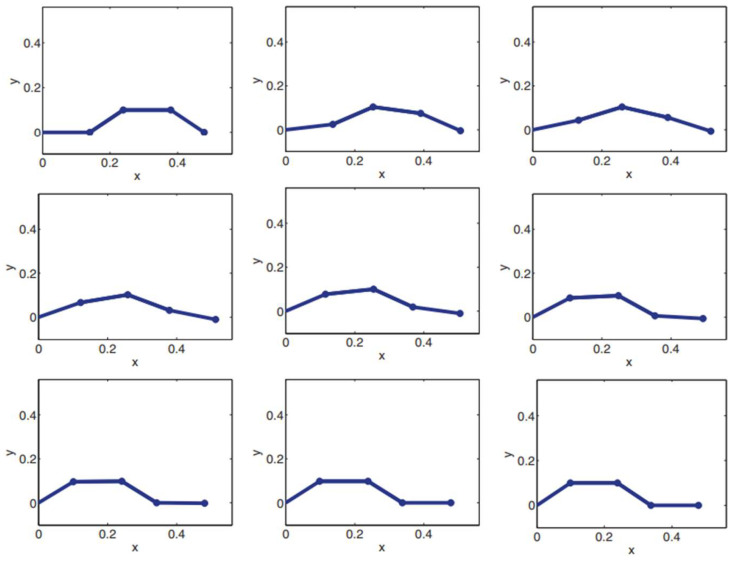
Simulation of the worm robot locomotion.

Figure 7 shows the simulation of a worm robot by using the proposed control method. In conclusion, the Koopman–LQR approach achieves excellent tracking and control performance, both in terms of joint positions and velocities. The ability to handle the nonlinear dynamics of the worm robot with minimal overshoot and smooth velocity transitions validates the suitability of the proposed control method for such complex systems. Friction is essential for the locomotion of worm robots because it facilitates grip and traction against the surfaces they traverse. This grip allows the robot to push against the ground effectively, converting its internal movements into forward motion. In worm robots, the design often includes specialized textures or materials at the tips to enhance frictional contact, which helps anchor the robot during movement cycles. This enables the body to contract and expand in a coordinated manner, producing a wave-like motion that propels it forward. Moreover, managing friction can also contribute to energy efficiency; too much friction can cause resistance and slow down movement, while too little can lead to slipping. By carefully calibrating these frictional forces, worm robots can achieve smoother, more controlled locomotion, allowing them to navigate complex terrains and obstacles with greater agility.

## 7. Conclusions

This paper presents the development and implementation of an optimal data-driven control strategy for a bio-inspired worm robot. The research begins with a detailed discussion of the nonlinear dynamic model governing the worm robot’s motion, emphasizing the complexities involved in accurately modeling its behavior due to its nonlinear characteristics. To address these challenges, Koopman operator theory is applied to transform the nonlinear dynamics into a linear representation, enabling the use of linear control techniques. The DMD method is employed to estimate the Koopman operator from the generated data of the worm robot’s dynamic behavior. This operator allows for an approximation of the nonlinear system as a linear model, facilitating more straightforward control design. Subsequently, an LQR controller is designed to regulate the worm robot’s joint movements. The LQR controller optimally balances the control effort and tracking accuracy by minimizing a predefined cost function. Simulation results demonstrate the effectiveness of the proposed Koopman–LQR control approach. Specifically, the results show that the controller achieves precise position tracking of the robot joints while ensuring smooth velocity profiles. These findings validate the performance of the proposed control scheme, highlighting its potential for real-time applications in worm robot control.

## Data Availability

Data are contained within the article.
